# Targeting of the circadian clock via CK1δ/ε to improve glucose homeostasis in obesity

**DOI:** 10.1038/srep29983

**Published:** 2016-07-21

**Authors:** Peter S. Cunningham, Siobhán A. Ahern, Laura C. Smith, Carla S. da Silva Santos, Travis T. Wager, David A. Bechtold

**Affiliations:** 1 Faculty of Life Sciences, University of Manchester, Manchester, UK; 2 Pfizer Worldwide Research and Development, Cambridge, MA, USA

## Abstract

Growing evidence indicates that disruption of our internal timing system contributes to the incidence and severity of metabolic diseases, including obesity and type 2 diabetes. This is perhaps not surprising since components of the circadian clockwork are tightly coupled to metabolic processes across the body. In the current study, we assessed the impact of obesity on the circadian system in mice at a behavioural and molecular level, and determined whether pharmacological targeting of casein kinase 1δ and ε (CK1δ/ε), key regulators of the circadian clock, can confer metabolic benefit. We demonstrate that although behavioural rhythmicity was maintained in diet-induced obesity (DIO), gene expression profiling revealed tissue-specific alteration to the phase and amplitude of the molecular clockwork. Clock function was most significantly attenuated in visceral white adipose tissue (WAT) of DIO mice, and was coincident with elevated tissue inflammation, and dysregulation of clock-coupled metabolic regulators PPARα/γ. Further, we show that daily administration of a CK1δ/ε inhibitor (PF-5006739) improved glucose tolerance in both DIO and genetic (*ob/ob*) models of obesity. These data further implicate circadian clock disruption in obesity and associated metabolic disturbance, and suggest that targeting of the clock represents a therapeutic avenue for the treatment of metabolic disorders.

Daily 24 hour rhythms are evident in virtually all aspects of our physiology including sleep/wake cycles, feeding behaviour, metabolism, and immune response^1^. In mammals, these rhythms are orchestrated by a master circadian clock housed within the suprachiasmatic nuclei (SCN) of the hypothalamus, and a coordinated network of semi-autonomous clocks operating throughout the brain and peripheral tissues of the body. The influence of these local tissue clocks extends well beyond circadian timing *per se*, having a profound impact on global patterns of gene expression, as well as overall tissue function^2^,^3^,^4^. The importance of the circadian system and specific components of the clock to human health has also become clear, with compelling evidence linking circadian disruption to many pathological conditions, including type 2 diabetes^5^. For example, conditions that undermine the clock, such as chronic sleep disturbance and forced desynchrony, disrupt glucose homeostasis and impair insulin sensitivity, while chronic shift-work is associated with an increased incidence of obesity and diabetes^6^,^7^,^8^,^9^.

The circadian clock is tightly and reciprocally coupled to energy metabolism at both a cellular and organism level. Transcriptional and proteomic profiling studies in mice and humans consistently show metabolic processes to be highly rhythmic and under clock control^10^,^11^,^12^,^13^,^14^,^15^. Moreover, the circadian clock itself is responsive to energy status, with the activity or expression of many clock constituents being influenced by major metabolic regulators, such as AMP-dependent kinase, sirtuins, and peroxisome proliferator-activated receptors α/γ (PPARα/γ)^1^,^16^,^17^,^18^,^19^,^20^. The reciprocal nature of this coupling allows both circadian and metabolic processes to adapt to natural feeding and fasting cycles, as well as shifts in the timing of food availability. For example, PPARα and PPARγ directly influence transcription of *Bmal1* and *Reverbα*^19^,^20^,^21^,^22^,^23^, and PPARα driven Rev-erbα expression is critical for realignment of the circadian clockwork to restricted feeding schedules in mice^24^. Of course, the close interconnection of clock and metabolic processes also implies that the circadian system may be compromised during metabolic dysfunction, such as during severe obesity, and thereby contribute to obesity-related pathology.

The impact of high fat diet (HFD) feeding on circadian rhythms in behaviour, as well as clock gene expression in central and peripheral tissues has been examined in a number of studies (e.g.^5^,^25^,^26^,^27^,^28^,^29^,^30^,^31^,^32^). However, the extent of HFD-induced disruption to the circadian system varies between reports, with minimal effects on clock gene expression reported in some cases^25^,^27^,^28^ and significant damping of circadian rhythms reported in others^29^,^32^. It also remains unclear whether altered clock function in DIO contributes to obesity-related metabolic dysfunction, such as insulin resistance. Here, we sought to clarify the impact of both acute and long-term HFD-feeding on the circadian system in mice. To this end, we undertook a broad analysis of behavioural and molecular rhythms in HFD-fed C57Bl/6J mice. Additionally, we examined whether targeting the clock via casein kinase 1δ/ɛ (CK1δ/ɛ) inhibition can confer metabolic benefit in obese mice. We have previously shown pharmacological targeting of CK1δ/ɛ to be effective in increasing SCN clock rhythms, and behavioural entrainment in otherwise arrhythmic mice^33^. Our current studies highlight a highly tissue- and gene-specific impact of HFD-feeding on the molecular clockwork, and show that pharmacological targeting of CK1δ/ɛ improves glucose tolerance in diet-induced and genetic models of obesity.

## Methods

### Animals

Experimental procedures were licensed under the 1986 Home Office Animal Procedures Act (UK), and approved by The University of Manchester animal welfare committee guidelines. All experimental procedures were carried out in accordance with the above licensing and guidelines. Male C57BL/6J and *ob/ob* mice were purchased from Charles River (UK) and Harlan (UK) respectively. mPER2::luc transgenic mice^34^ were kindly provided by Joe Takahashi (University of Texas Southwestern) and subsequently bred locally. All animals were maintained in 12 h:12 h light:dark (LD) with an ambient temperature of 20–22 °C, with food and water supplied *ad libitum*.

### Diet-induced obesity and assessment of physiological rhythms

For DIO studies, 8–10 week old mice were provided *ad libitum* access to high fat diet (HFD; 60% energy from fat; DIO Rodent Purified Diet, IPS Ltd) or normal chow (NC) for 2, 8, or 16 weeks. Prior to tissue collection, mice were placed in constant darkness (DD) and tissues collected every 4 h (n = 5–10/time-point/diet condition). In selected experiments, body temperature (T_b_) and locomotor activity were recorded using radiotelemetry (Data Sciences International), and metabolic gas exchange measured by indirect calorimetry using the CLAMS system (Columbus Instruments)^35^. Food intake was monitored using the Labmaster Metabolism Research Platform (TSE systems), with meal size and feeding events (see Supplementary Fig. S1) recorded over 6 days. For *ob/ob* studies, mice were maintained on NC throughout, and locomotor activity was recorded in home cages using infrared beam-break sensors.

### Drug administration and glucose tolerance testing (GTT)

DIO mice (16 weeks of HFD) and matched NC-fed controls were injected daily with vehicle (20% 2-hydroxypropyl β-cyclodextrin; Sigma) or the selective inhibitor of CK1δ/ε, PF-5006739 (10 mg/kg/day, s.c.) at ZT10. This dose of PF-5006739 and timing of administration was based on previous work, and selected to achieve a CNS target occupancy above 50% for CK1δ/ε^36^ (Supplementary Table S1). After 3 weeks of drug treatment, mice were fasted for 6 h before administration of glucose (1 g/kg i.p., n = 7–10/group). Blood glucose was monitored with an Accu-chek Aviva glucose meter (Roche), and serum samples collected at 0 and 30 min post-glucose administration. Mice were then maintained on PF-5006739/vehicle dosing for a further 14d prior to tissue collection. For *ob/ob* studies, mutant and control mice (8 weeks of age) were dosed PF-5006739 as described above for 14d, following which a GTT was performed (20 h fast followed by 1 g/kg glucose i.p). Serum insulin and adiponectin levels were determined by ELISA (Millipore and R&D Systems, respectively).

### *In situ* hybridisation

*In situ* hybridisation was performed as previously described^37^. Briefly, brains were rapidly dissected and frozen. Coronal sections (12 μm) were collected using a cryostat freezing microtome, and stored at −80 °C until processing. The *Per1* probe was kindly provided by Prof. Urs Albrecht (University of Fribourg). *Bmal1* and *Rev-erbα* probes (primers listed in Supplementary Table S2) were cloned into pGEM-T easy. All probes were synthesized in the presence of ^33^P-uridine triphosphate (MP Biomedicals). Hybridization was visualized by film autoradiography (Kodak BioMax MR film), and OD determined using 3–4 sections per mouse, and 5 mice per time-point/group.

### Quantitative real-time PCR (qPCR) and Western blot analysis

Peripheral tissues were rapidly dissected, snap frozen, and stored at −80 °C until use. Hypothalamic blocks were micro-dissected from 300 μm coronal brain slices. RNA extraction and qPCR was performed as previously described^38^ (qPCR primers listed in Supplementary Table S2). For protein extraction, frozen tissues were homogenised in Tissue-Protein Extraction Reagent (Peirce) containing Protease Inhibitor Cocktail (Roche) and 1 mM PMSF. Protein levels were determined by SDS-PAGE and Western blot analysis using anti-PPARγ (C26H12, Cell Signalling, 1:500) and anti-GAPDH (FL-335, Santa Cruz Biotechnology, 1:1000) antibodies.

### Bioluminescence Recording

For *ex vivo* analyses of circadian rhythms, 8 week old male mPER2:luc mice were fed HFD or NC for 16 weeks. mPER2-dependent bioluminescence was recorded from gWAT samples maintained at 37 °C using a Lumicycle (Actimetrics) as described previously^33^. Amplitude and phase of the second peak post-culture was determined for each animal (n = 4 mice/condition; 16 samples/mouse). To determine the effect of PF-5006739 on mPER2::luc expression, cultured gWAT were treated with vehicle (water) or PF-5006739 (0.08–50 μM), 5d post-culture. Amplitude was measured at the first peak post-treatment using Lumicycle recording, or bioluminescence pre- and 12 hr post-treatment using a GloMax-Multi Luminometer (Promega).

### Statistical analysis

Data are presented as mean ± standard error (SEM). Students’ t-test was used when two groups were tested, one-way ANOVA with Dunnett’s multiple comparisons or two-way ANOVA followed by Sidak post-hoc analyses were used when more than two groups and/or factors were analysed. Statistical determination of acrophase was performed by linear harmonic regression using CircWave v1.4 software^39^.

## Results

### Behavioural rhythmicity in obese mice

**Figure 1:**
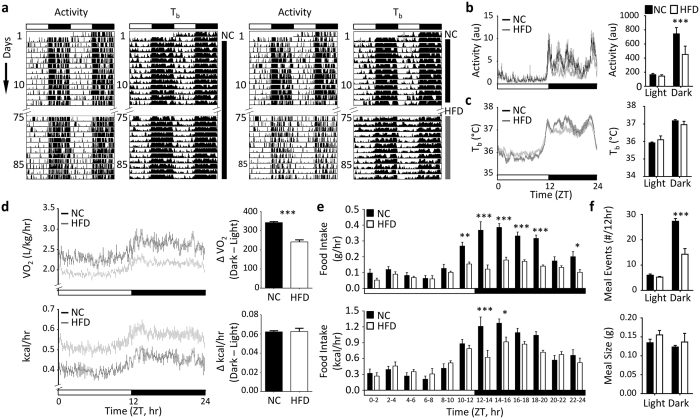
Behavioural rhythmicity in diet-induced obese mice. (**a–c**) Robust diurnal rhythms of locomotor activity (**a**,**b**) and body temperature (T_b_; **a**,**c** were maintained in NC and HFD-fed mice throughout the study (n = 7/group); although a reduction in night-time activity was observed in the HFD-fed mice (**b**). (**d**) Indirect calorimetry revealed an obesity-related decrease in oxygen consumption (VO_2,_ relative to body weight) and increase in energy consumption (kcal/hr/mouse). A significant reduction in day-night amplitude was evident in VO_2_ profiles of HFD-fed mice. (**e,f**) Analysis of feeding behaviour revealed a significant reduction in food intake (**e**) and feeding events (**f**) in HFD-fed mice specifically during the dark phase of the cycle. Individual meal size was unaffected by HFD feeding. Data reflect mean ± SEM; *p < 0.05, **p < 0.01, ***p < 0.001; (**b,c**,**f**) two-way ANOVA with Sidak post hoc test, (**e**) repeated-measures two-way ANOVA and (**d**) Student’s t-test.

To assess the impact of diet-induced obesity (DIO) on behavioural and physiological rhythms, adult male C57BL/6J mice were maintained on NC or HFD (60% energy from fat) for 16wk, leading to pronounced weight gain and WAT accumulation (Supplementary Fig. S1). Diurnal rhythms in locomotor activity and body temperature (T_b_) were maintained in HFD-fed and control mice throughout the 16wk study (Fig. 1a–c). However, a reduction in the amplitude of activity and T_b_ between light-dark periods was observed in the obese mice, due principally to a reduction in night-time activity and increased day-time T_b_. Assessment of metabolic gas exchange at the end of the study period (16wk HFD) showed a significant reduction in overall rates of oxygen consumption (VO_2_, L/kg/h) and increased rates of energy consumption (kcal/h) in DIO mice (Fig. 1d). In line with activity and T_b_ measures, diurnal rhythmicity in metabolic gas exchange was maintained, but with a significant reduction in diurnal amplitude. Increased daytime feeding is commonly reported in HFD-fed mice, and we therefore assessed diurnal feeding structure in mice fed NC or HFD. The proportion of calories consumed during the light phase of the cycle was increased upon HFD feeding (Supplementary Fig. S1). However, this reflected a decrease in food intake during the dark phase, in terms of overall consumption and meal frequency, rather than increased feeding in the day (Fig. 1e,f). Notably, altered nocturnal feeding was observed within days of the mice being placed on HFD (Supplementary Fig. S1), likely reflecting a homeostatic response to the increased calorific and fat content of the diet, rather than an obesity-dependent modulation of circadian feeding rhythms.

Although these studies demonstrate that diet-induced obesity did not overtly disrupt diurnal rhythmicity in the mice or their ability to entrain to the LD cycle, the relative amplitudes of physiological measures were reduced in HFD-fed mice when compared to NC-fed controls.

### Disruption of the molecular clockwork in response to obesity

**Figure 2:**
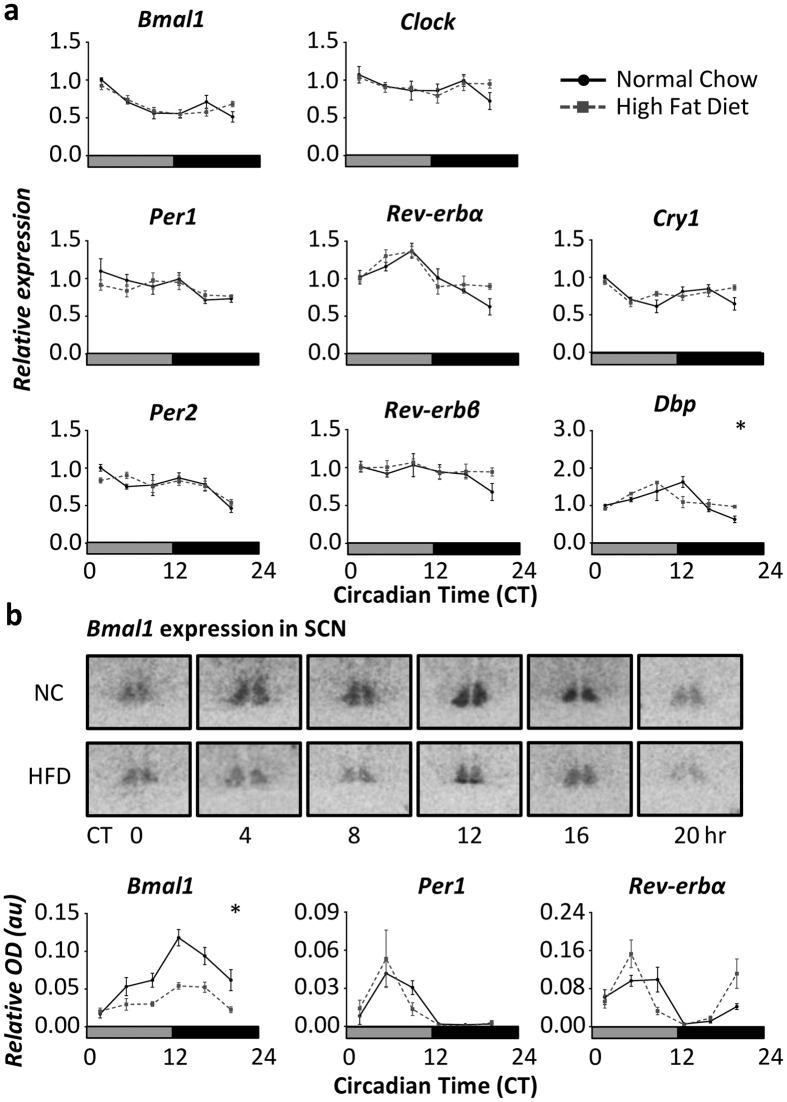
Impact of diet induced obesity on hypothalamic clock gene expression. (**a**) Analyses of gene expression in microdissected hypothalamic tissue revealed that rhythmic expression of clock genes was largely unaffected by long term HFD feeding (n = 5/diet/time-point). (**b**) However, analyses of the SCN by *in situ* hybridisation revealed a significant damping of *Bmal1* expression in DIO mice (n = 5mice/diet/time-point with 3–4 SCN sections/mouse; representative autoradiograph images shown). *Per1* and *Rev-erbα* rhythms in the SCN of DIO mice were advanced in phase relative to NC-fed mice, but showed no evidence of damped expression. *Indicates a significant difference between NC and HFD profiles (p < 0.01, two-way ANOVA with Sidak post hoc test) at one or more time-points. Data reflect mean ± SEM. Samples were collected under DD, and therefore the grey bar on the x-axes represents subjective day.

We next assessed the impact of obesity on clock gene rhythms in a panel of central and peripheral tissues collected from mice fed HFD or NC for 16 weeks. Gene expression analyses of micro-dissected hypothalamic blocks did not reveal any significant alteration in expression of the core clock genes *Bmal1, Clock, Per1, Per2, Cry1, Rev-erbα* and *Rev-erbβ* (Fig. 2a). As these blocks contain a number of oscillatory sites aside from the SCN (e.g. arcuate and dorsomedial nuclei), we employed *in situ* hybridisation to assess clock gene rhythms in the SCN. *In situ* hybridization analyses revealed a significant damping of *Bmal1* expression in the SCN of DIO mice when compared to matched NC-fed controls (Fig. 2b). SCN expression of *Bmal1* remained rhythmic in the DIO mice, yet exhibited a reduced level of expression relative to NC-fed mice at all time-points and reduced amplitude overall. Despite the damping of *Bmal1* expression, *Per1* and *Rev-erbα* expression within the SCN was similar between DIO and control mice, although both genes exhibited a small advance in acrophase within the DIO mice (Fig. 2a, Supplementary Fig. S2).

**Figure 3:**
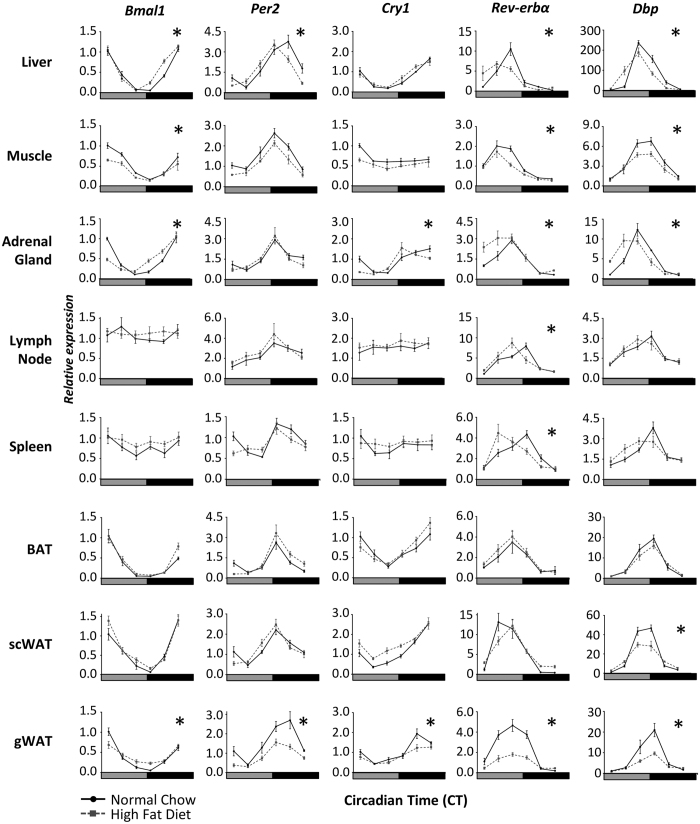
Tissue-specific disruption of the molecular clock in obese mice in peripheral tissues. Clock gene expression profiles in peripheral tissues were compared between mice fed NC or HFD for 16wk (n = 5/diet/time-point). Differences in gene expression were observed at a number of individual time-points. For clarity, *Indicates a significant difference between NC and HFD profiles (p < 0.01, two-way ANOVA with Sidak post hoc test) at one or more time-points within a specific tissue profile. The amplitude of clock gene expression was largely unaffected by long term HFD feeding in all tissues except for gWAT, where a significant attenuation of *Bmal1, Per2, Cry1, Rev-erbα* and *Dbp* expression was observed. Advanced gene rhythms were observed in the liver and adrenal gland. Additional gene profiles and acrophase analyses are shown in Supplementary Fig. S2 and Table S3. Data reflect mean ± SEM. Samples were collected under DD, and grey bar on the x-axes represents subjective day.

In peripheral tissues, analysis of circadian clock gene expression in HFD- and NC-fed mice revealed that robust rhythms were maintained in most tissues examined (Fig. 3, Supplementary Fig. S2). However, a pronounced alteration of clock gene expression was observed in perigonadal WAT (gWAT). Interestingly, clock gene expression was relatively unaffected in scWAT of obese mice, suggesting that reduced amplitude in gWAT was not due simply to increased expansion and triglyceride storage within the adipose tissues. The differential impact of obesity on clock gene expression in the gonadal and subcutaneous adipose depots was most pronounced in *Rev-erbα, Per1* and *Per2* gene expression profiles. The damping of gWAT rhythms in response to chronic HFD-feeding was maintained *ex vivo*, in tissue explants derived from 16wk HFD-fed mPER2::luciferase reporter mice, when compared to tissue derived from NC-fed matched controls (Supplementary Fig. S2). In addition to damping of gWAT clock gene expression, evidence of altered phase alignment between tissue clocks was also observed in HFD-fed mice. Specifically, a phase advance of transcriptional rhythms was observed in the liver and adrenal gland of HFD-fed mice (~2.0 h phase advance in HFD relative to NC-fed, based on acrophase of the 8 clock genes profiled; Supplementary Table S3), but not other tissues such as skeletal muscle and BAT, indicating reduced synchrony among tissue oscillators. Throughout the peripheral tissue panel, the most profoundly and consistently affected transcript was *Rev-erbα*, which showed no significant time of day difference in expression across the circadian cycle in gWAT of HFD-fed mice (Fig. 3). Interestingly, we did not observe a similar effect in the related gene, *Rev-erbβ*, which was largely unaffected by HFD feeding across the eight peripheral tissues examined in this study (Supplementary Fig. S2). This highlights the differential regulation of the two related nuclear hormone receptors.

Overall, these studies demonstrate a pronounced effect of chronic HFD feeding on expression and synchrony of the molecular clockwork, and highlight the gene- and tissue-specificity of these effects.

### gWAT clock damping associated with reprograming of clock-metabolic regulators

**Figure 4:**
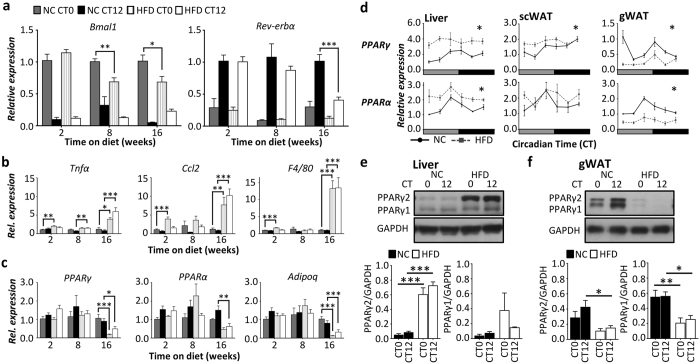
Chronic HFD feeding is associated with adipose tissue inflammation and alteration in PPAR expression. (**a**) Damping of clock gene expression in gWAT was associated with long-term HFD-feeding (16wk), rather than an acute response to 2wk or 8wk of HFD (n = 5–6/group). (**b**) Assessment of inflammatory gene expression following 2, 8 and 16wk of HFD-feeding revealed that development of inflammation in gWAT was most pronounced following 16wk HFD feeding. (**c**) Similarly, attenuated expressions of the metabolic regulators *PPARγ, PPARα*, and the PPARγ-regulated gene *Adipoq* was observed following 16 weeks on HFD, but not after 2 or 8 weeks. (**d**) At 16wk, obesity-related alteration in *PPARγ* and *PPARα* expression was highly tissue specific. Both receptors exhibited increased expression in the liver, yet profoundly attenuated expression in gWAT of HFD-fed mice compared to matched WT mice (n = 5/diet/time-point). (**e**) Similarly, PPARγ protein expression exhibited a dramatic and tissue-specific response to long-term HFD feeding (liver n = 3/group, gWAT n = 6/group). Data expressed as mean ± SEM; (**a–c**,**e**) two way ANOVA with Sidak post hoc test; *p < 0.05, **p < 0.01, ***p < 0.001; (**d**) *indicates a significant difference between diets at one or more time-points; p < 0.01, two-way ANOVA with Sidak post hoc.

To further assess the impact of HFD-feeding on clock gene expression, mice were maintained on NC or HFD for 2, 8, or 16 wk (Fig. 4a–c, Supplementary Fig. S3). Importantly, damping of *Bmal1* and *Rev-erbα* expression in gWAT was not observed following 2wk of HFD-feeding, and was most pronounced in mice that had been maintained on HFD for 16wk (Fig. 4a). Therefore, damping of clock gene rhythms in gWAT of obese mice was not due to an acute effect of the diet. In animals and humans, obesity-related insulin resistance has been linked to increased immune cell infiltration and pro-inflammatory cytokine production within hypertrophic WAT^40^. We therefore profiled pro-inflammatory markers in the tissues of NC and HFD-fed mice. A profound elevation of gWAT inflammation was observed in 16wk HFD-fed mice compared with matched mice fed NC, and those fed HFD for 2wk or 8wk (Fig. 4b). At 16wk of HFD-feeding, expressions of the pro-inflammatory cytokines *Ccl2* and *TNFα*, as well as the macrophage marker *F4/80* were most profoundly elevated in gWAT (Supplementary Fig. S3), suggesting that clock gene damping within this tissue may be linked to the development of obesity-related inflammation.

Components of the circadian clock are also closely and reciprocally linked with the metabolic regulators, PPARα and PPARγ^5^,^19^,^41^. We therefore examined tissue-specific and temporal dynamics of PPARα/γ expression in mice maintained on HFD (Fig. 4c–f). Similar to the attenuation of clock gene rhythms, a significant reduction in *PPARα/γ* expressions were observed in gWAT of mice maintained on HFD for 16wk (but not 2wk or 8wk; Fig. 4c). Obesity-related changes in *PPARα* and *PPARγ* expression were highly tissue specific, with the expressions of both receptors significantly increased in the liver, yet profoundly attenuated in gWAT of obese mice when compared with matched NC-fed controls (Fig. 4d). In contrast to gWAT, *PPARα* and *PPARγ* expressions were not reduced in scWAT (Fig. 4d). Tissue-specific changes in *PPARγ* mRNA expression in liver and gWAT were mirrored in PPARγ protein expression (Fig. 4e,f), as well as in PPAR*γ* target genes including *Adipoq* and *Fabp4* (Fig. 4c, Supplementary Fig. S3), and genes involved in lipid metabolism known to be under circadian control (Supplementary Fig. S3).

These studies reveal a pronounced tissue-specific nature of obesity-related clock dysfunction, with gWAT being particularly affected. However, our findings also highlight the fact that damping of the clock in gWAT is accompanied (in terms of duration of HFD-feeding and tissue-specificity) by elevated tissue inflammation and altered *PPARα*/*γ* expression.

### Targeting of the clock by CK1δ/ε inhibition

**Figure 5:**
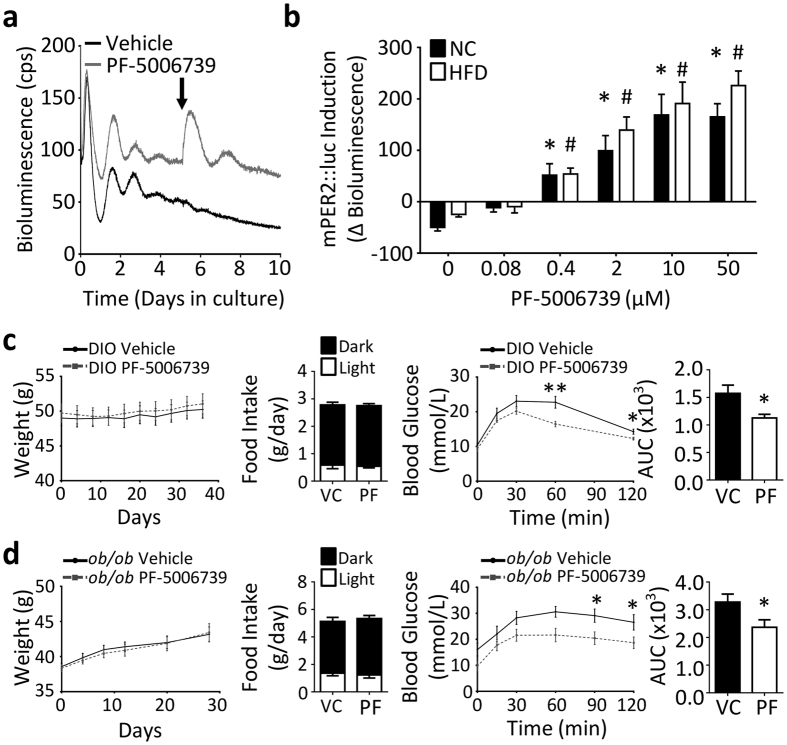
Pharmacological targeting of the clock via PF-5006739 improves glucose tolerance. (**a**) Representative recordings of mPER2::luc driven bioluminescence from gWAT explants, where treatment of tissue with PF-5006739 (0.4 μM, arrow) caused a rapid and robust induction of bioluminescence. (**b**) The induction of gWAT mPER2::luc bioluminescence by PF-5006739 was dose-dependent and effective in tissues derived from NC and HFD-fed mice. Data reflects the change in mPER2::luc bioluminescence from time of treatment to 12 hr post-PF-5006739 application (n = 4/diet/dose). (**c,d**) Daily dosing with PF-5006739 (10 mg/kg/day, at ZT10) was performed in DIO mice (**c**, n = 13/group) and ob/ob mice (**d**, n = 10/group). Treatment with vehicle (VC) or PF-5006739 (PF) did not alter body weight or food intake in the mice. However, a significant improvement in glucose tolerance was observed in both DIO and *ob/ob* mice treated with PF-5006739 when compared with vehicle treated mice (GTT, n = 7/group). Data reflect mean ± SEM; (**b**) *p < 0.05 vs NC 0 μM, ^#^p < 0.05 vs HFD 0 μM; two-way ANOVA with Dunnett’s multiple comparisons vs 0 μM. (**c**,**d**) *p < 0.05, **p < 0.01, repeated measures one-way ANOVA (GTT profile) and Student’s t-test (area under the curve; AUC).

We have previously shown that daily administration of CK1δ/ε inhibitors can be effective at increasing the amplitude of disrupted and weak oscillators^33^. We therefore assessed whether this approach would be effective in DIO mice using a novel CK1δ/ε inhibitor (PF-5006739), which exhibits improved target selectivity over previous compounds^36^. The impact of CK1δ/ε inhibition on damped rhythms was first examined using gWAT tissue cultures derived from mPER2:Luc mice which had been maintained on NC or HFD for 16wk (Fig. 5a,b). WAT tissue explants were maintained in culture for 5 days, and then treated with vehicle or PF-5006739 (0.4–50 uM). In gWAT tissue derived from either NC or HFD-fed mice, PF-5006739 administration caused a rapid and dose-dependent induction of mPER2::luc bioluminescence (Fig. 5a,b).

We next tested whether pharmacological targeting of CK1δ/ε could provide a metabolic benefit *in vivo* in models of obesity. DIO mice (16wk HFD-feeding) were treated once daily at ZT10 with PF-5006739 (10 mg/kg/day). Dose and timing of administration were based on prior characterisation of the compound^36^. Following 3 weeks of treatment, no significant impact of PF-5006739 was observed in NC or HFD-fed mice (relative to vehicle-treated mice for the same diet group) with respect to body weight, food intake, day/night feeding behaviour, or diurnal activity profile (Fig. 5c, Supplementary Fig. S4). However, daily administration of the CK1δ/ε inhibitor did lead to a significant improvement in glucose tolerance in response to bolus glucose challenge (glucose tolerance test; GTT) (n = 7/group; Fig. 5c). Damping of circadian gene rhythms in peripheral tissues has been reported in leptin-deficient *ob/ob* mice^30^, a genetic model of obesity. We therefore tested the ability of PF-5006739 administration to improve glucose tolerance in this model. Similar to studies in DIO mice, PF-5006739 treatment (10 mg/kg/day, ZT10) did not affect body weight, feeding behaviour, or activity profiles in *ob/ob* mice, relative to vehicle-treated *ob/ob* mice (Fig. 5d, Supplementary Fig. S4). Yet once again, daily administration of PF-5006739 significantly improved blood glucose profiles during GTT (Fig. 5d). No difference in glucose clearance was observed in lean (NC-fed) C57Bl/6J mice treated with PF-5006739 relative to vehicle-treated mice (Supplementary Fig. S4).

Taken together these studies highlight the potential benefits of targeting CK1δ/ε to improve glucose homeostasis in obesity.

## Discussion

The studies detailed here contribute to the growing evidence that the molecular clockwork is compromised during obesity. Our studies highlight obesity-related desynchrony and a pronounced disruption of the circadian clockwork in visceral WAT. Attenuated clock gene rhythms in gWAT were not due to altered nutritional input (i.e. acute consumption of HFD), but were associated with chronic HFD-feeding and dysregulation of PPARα/γ. Further, we show that pharmacological targeting of CK1δ/ε, potent modulators of the molecular clock, was effective in improving glucose tolerance in both diet-induced and genetic models of obesity.

The impact of HFD feeding on circadian rhythms in behaviour, as well as clock gene expression in central and peripheral tissues has been examined in a number of studies (e.g.^5^,^25^,^26^,^27^,^28^,^29^,^30^,^31^,^32^). However, the extent to which HFD-feeding impacts the circadian system varies between these reports. These discrepancies are perhaps not unexpected, as we have shown here that the duration of HFD feeding has a significant impact, and that alterations to the phase and amplitude of peripheral clock gene expression are tissue and gene specific. Here, across two large independent studies, we reveal clear evidence of both damped rhythms (most notably in gWAT, and *Bmal1* expression in the SCN), and tissue desynchrony (i.e. advanced rhythms in liver and adrenal gland, but not in muscle, BAT and scWAT) which appear in response to long-term HFD-feeding in DIO mice. Our studies also highlight the damping of numerous physiological rhythms (T_b_, calorie intake, locomotor activity, VO_2_) across the diurnal cycle in the HFD-fed mice, as well as a significantly damping of circadian expression of *Bmal1* within the SCN. We have not established whether damped *Bmal1* expression in the SCN contributes directly to the damping of physiological rhythms, but it does demonstrate a clear impact of HFD-feeding on the master circadian clock. Moreover, timing of food intake, temperature, and activity are well-established and potent entrainment cues for peripheral tissue clocks; thus, the reduced amplitude of physiological rhythms (T_b_, activity) and HFD-induced shift in calorie intake towards the inactive phase of the cycle represents a pronounced damping of these internal zeitgebers in the obese animals. Advancement of hepatic clock gene rhythms has been reported previously in HFD-fed mice, in response to acute changes in diet composition^31^,^42^, suggesting desynchrony may not be due to obesity *per se* but rather in response to altered dietary intake.

An interesting difference in the susceptibility of visceral and scWAT clocks to obesity-related dysfunction was observed in our studies. This has been suggested in a previous study, although no data was provided^32^. Adipose depot specific differences in the response of the circadian clock to obesity has also been reflected in human studies^43^,^44^. In line with previous work^29^,^31^,^45^, the expressions of *PPARα* and *PPARγ* were significantly altered in the mice by high fat diet feeding. Our studies highlight the association (in terms of timing and tissue-specificity) of obesity-related clock gene disruption with altered PPARα and PPARγ expression and local tissue inflammation. Expression of PPARα and PPARγ are influenced by the clock, but both receptors also regulate directly the expression of clock components; most notably, *Rev-erbα* is a well established target of PPARγ^1^,^21^,^22^. Here, WAT depot-specific differences in PPARα and PPARγ expression in response to DIO exhibit a similar profile to *Rev-erbα* expression in those tissues, suggesting that alterations in PPAR and clock gene expression observed in the obese mice may be directly linked. Interestingly, inducible deletion of PPARγ in adult mice^46^ leads to a significant damping of *Per1* and *Rev-erbα*, but not *Per2* or *Cry1* within adipose tissue; a gene specificity similar to that observed in the gWAT of the obese mice in our studies. The significant role of PPARα and PPARγ in circadian and metabolic adaption of the liver transcriptome in response to altered diet and timing of food intake has been clearly demonstrated^24^,^31^,^47^. For example, HFD-feeding induced expression of PPARγ in the liver orchestrates a dramatic reorganisation of rhythmic expression of metabolic genes^31^. Therefore, the pronounced tissue-specific (e.g. liver vs gWAT) change in *PPARα* and *PPARγ* expression observed here in the DIO mice suggests an alteration of transcriptional rhythmicity across metabolic tissues, which reflect a more profound temporal desynchrony than indicated by the clock gene expression.

The detrimental impact of clock disruption on energy homeostasis and insulin signalling is evident in both animal and human studies^3^,^48^. For example, increased susceptibility to diet-induced obesity, altered insulin signalling, hyperglycemia and/or glucose intolerance have been reported in a number of clock gene mutant or knockout mouse lines (e.g.^5^,^41^,^49^). In humans, desynchrony studies demonstrate that misalignment of internal clock rhythms from behavioural routine leads to disrupted glucose homeostasis and insulin sensitivity^6^,^9^,^50^. These studies implicate a role for circadian disruption and desynchrony in the metabolic disturbances associated with obesity, and suggest that strengthening of the clock can confer benefit. Indeed, pharmacological targeting of Rev-erb has been shown to reduce adiposity and improve glucose tolerance in obese mice^51^. Here, we demonstrate that daily administration of PF-5006739 similarly improves glucose tolerance. Although we did not profile circadian clock gene expression across the cycle in drug-treated mice in the current study, we have previously shown that CK1δ/ε inhibition can increase the amplitude of disrupted SCN rhythms *in vitro* and establish consolidated behavioural activity rhythms in otherwise arrhythmic mice^33^. Similarly, in the current study, PF-5006739 enhanced mPER2::Luc bioluminescence in damped adipose tissue explants. Further, altered glucose profiles were not observed in NC-fed mice treated with PF-5006739, and GTT studies were conducted well beyond the *in vivo* clearance time of the drug^36^. This suggests that it is a chronic effect of daily PF-5006739 administration (for example via enhanced clock entrainment), rather than a direct effect of CK1δ/ε inhibition *per se* that led to improved glucose tolerance in the obese mice. We suggest that daily targeting of the clock via CK1δ/ε inhibition in the obese mice underlies the improved glucose tolerance in the animals. Nevertheless, CK1δ and ε are known to regulate a variety of cellular processes outside of the circadian clock^52^, and therefore involvement of non-circadian pathways in mediating PF-5006739 improved glucose tolerance must also be considered. For example, previous work has implicated CK1δ in the regulation of hepatic gluconeogenesis via PGC-1α phosphorylation^53^, and glucose uptake into cultured adipocytes^54^.

In summary, this work highlights the tissue- and gene-specific clock disruption that accompanies diet-induced obesity. Importantly, daily administration of a highly selective CK1ε/δ inhibitor, which was effective at enhancing clock gene expression, led to an improved glucose tolerance in DIO and *ob/ob* mice. These studies provide further evidence of the widespread effects of high fat diet on the molecular clockwork, and reinforce the circadian clock as a novel avenue to achieve benefit in metabolic diseases, including obesity and type 2 diabetes.

## Additional Information

**How to cite this article**: Cunningham, P. S. *et al*. Targeting of the circadian clock via CK1δ/ε to improve glucose homeostasis in obesity. *Sci. Rep.* **6**, 29983; doi: 10.1038/srep29983 (2016).

## Supplementary Material

Supplementary Information
