# Supplementary material for: Targeting of the circadian clock via CK1δ/ε to improve glucose homeostasis in obesity
Source: Sci Rep. 2016 Jul 21;6:29983. doi: 10.1038/srep29983 (PMC4954991; doi:10.1038/srep29983)
Supplement: Supplementary Information [file srep29983-s1.pdf]

# Targeting of the circadian clock via CK1δ/ε to improve glucose homeostasis in obesity

Peter S. Cunningham, Siobhán A. Ahern, Laura C. Smith, Carla S. da Silva Santos, Travis T. Wager and David A. Bechtold

Supplementary Information

# Supplementary Figure Legends

**Supplementary Figure S1**. **a)** Individual body weights following 16wk feeding on normal chow (NC) or high fat diet (HFD) (n=30). Data reflect mean ± SEM; *** = p<0.001, Student's t-test. **b)** Pronounced WAT accumulation in diet-induced obese (DIO) mice (n=7-30). Data reflect mean ± SEM; *** = p<0.001, Student's t-test. **c)** Food intake during the light phase was increased following long term (10wk) HFD feeding (n=6-8 group-housed cages). Data reflect mean ± SEM; *** = p<0.001, Student's t-test. **d)** Food intake recorded in 10 minute bins for NC-fed and HFD-fed mice showing diurnal feeding structure. Reduced night-time feeding occurred within a few days of HFD feeding; although individual meal size (g/bin) was unaffected by HFD feeding. Dashed line indicates 0.05g/10min used to define feeding bouts.

**Supplementary Figure S2**. **a)** Representative autoradiograph images of *Per1* and *Rev-erbα in situ* hybridisation in the SCN at peak (CT4) and trough (CT16) of gene expression. Both genes exhibited a small advance in acrophase within the DIO mice (*Per1,* 1.3hr; *Rev-erbα,* 2.8hr) **b)** Expression profiles in peripheral tissues of the clock genes *Clock, Per1 and Rev-erbβ* (n=5/diet/time-point). Differences in gene expression were observed at a number of individual time-points. For clarity, * indicates a significant difference between NC and HFD profiles (p<0.01, two-way ANOVA with Sidak post hoc test) at one or more time-points within a specific tissue. Data reflect mean ± SEM. Samples were collected under DD, and therefore the grey bar on the x-axes represents subjective day. **c)** Damping of the gWAT molecular clock. *Ex vivo* analysis of gWAT tissue from mPER2::luc male mice fed NC or HFD (n=4/diet) showed decreased amplitude and a phase advance of mPER2::luc oscillation in DIO mice (Student's t-test). The mPER2::luc amplitude (counts per second, cps) was determined for each animal as the difference between the 1st nadir and 2nd peak using the mean of multiple tissue explants (n=16 samples/mouse).

**Supplementary Figure S3**. **a)** Expression of inflammatory markers in the liver, gWAT and scWAT following 16wk of HFD-feeding. The magnitude of inflammatory gene expression (especially of the macrophage marker *F4/80*) was most pronounced in the gWAT of HFD-fed mice (n=30/group). Data reflect mean ± SEM; *** = p<0.001, student's t-test. **b)** The PPAR target gene *Fabp4* showed differential regulation in the liver and WAT following 16wk HFD-feeding (n=5/diet/time-point; * indicates a significant difference between diets at one or more time-points; p<0.01, two-way ANOVA with Sidak post hoc). **c)** Genes involved in lipid metabolism (*Dgat2, Fasn, Atgl, Hsl* and *Lpl)* and glucose metabolism (*Pepck, Glut4, Glucokinase and Hexokinase 2 (Hk2))* were measured in the liver, scWAT and gWAT of mice fed HFD for 16wk. In gWAT, HFD feeding caused reduced expression of *Dgat2, Fasn, Atgl and Hsl,* with damping of the circadian expression of *Dgat2* and *Fasn.* In contrast, *Dgat2, Hsl* and *Lpl* were upregulated in scWAT (n=5/diet/time-point; * indicates a significant difference between diets at one or more time-points; p<0.01, two-way ANOVA with Sidak post hoc). **d)** The expression of *PPARγ* and *PPARα* was measured in the liver following 2, 8, and 16 wks of HFD feeding (n=5-6/group). Data are mean ± SEM; * = p<0.05, ** = p<0.01wo way ANOVA with Sidak post hoc.

**Supplementary Figure S4**. **a)** No significant impact of PF-5006739 on body weight, food intake, or glucose tolerance (glucose, 2g/kg; n=8/group) was observed in NC-fed mice. **b)** 24hr activity profile of DIO mice (% of daily wheel running activity, n=7/group) and *ob/ob* mice (% of daily locomotor activity, n=10/group) treated with either vehicle control or PF-5006739 (10mg/kg/day at ZT10). Arrow indicates time of dosing. **c)** Circulating glucose, insulin and adiponectin parameters in DIO and *ob/ob* mice treated with vehicle or PF-5006739. Data are mean ± SEM.

# Supplementary Figure S1

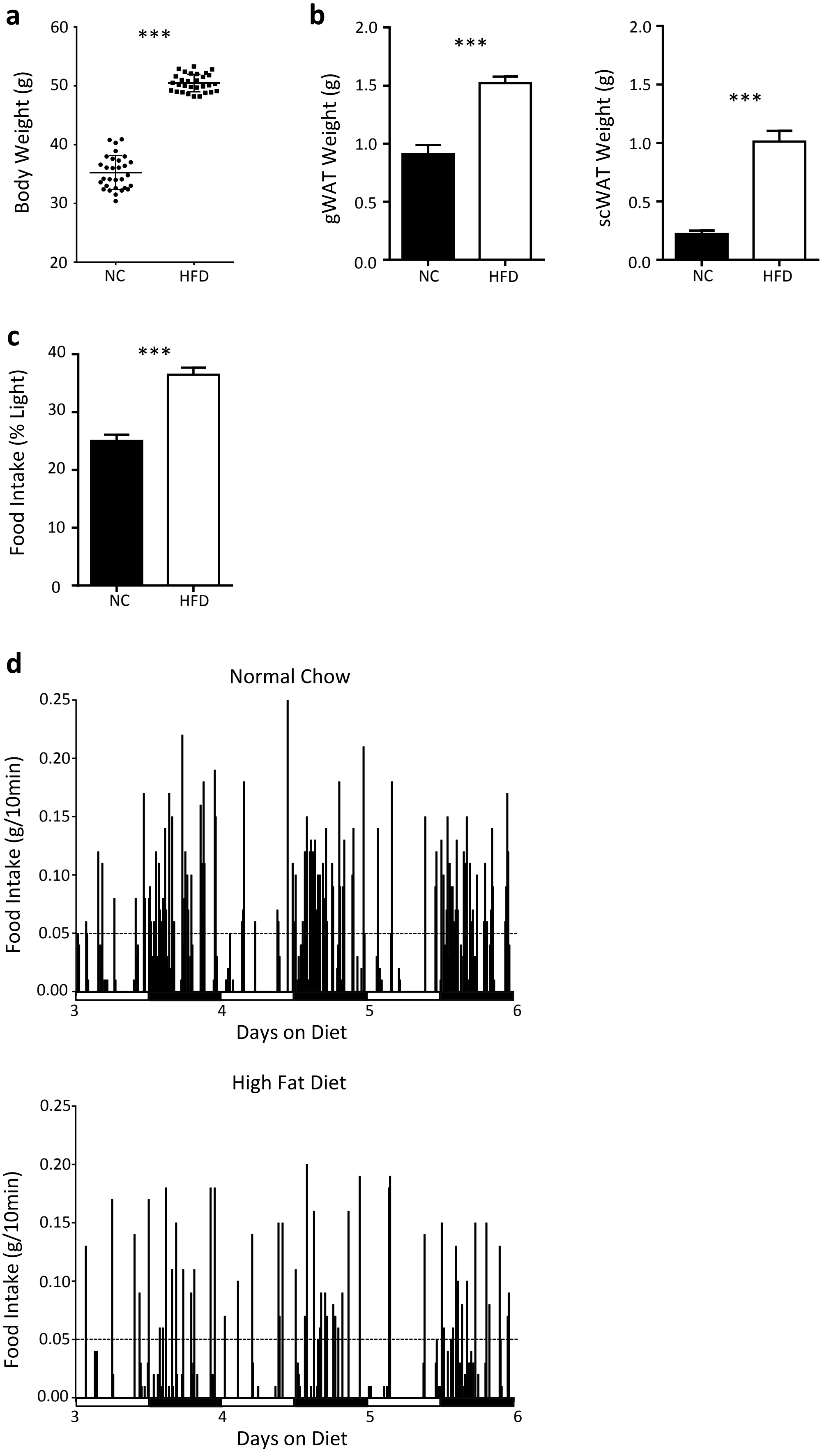

# Supplementary Figure S2

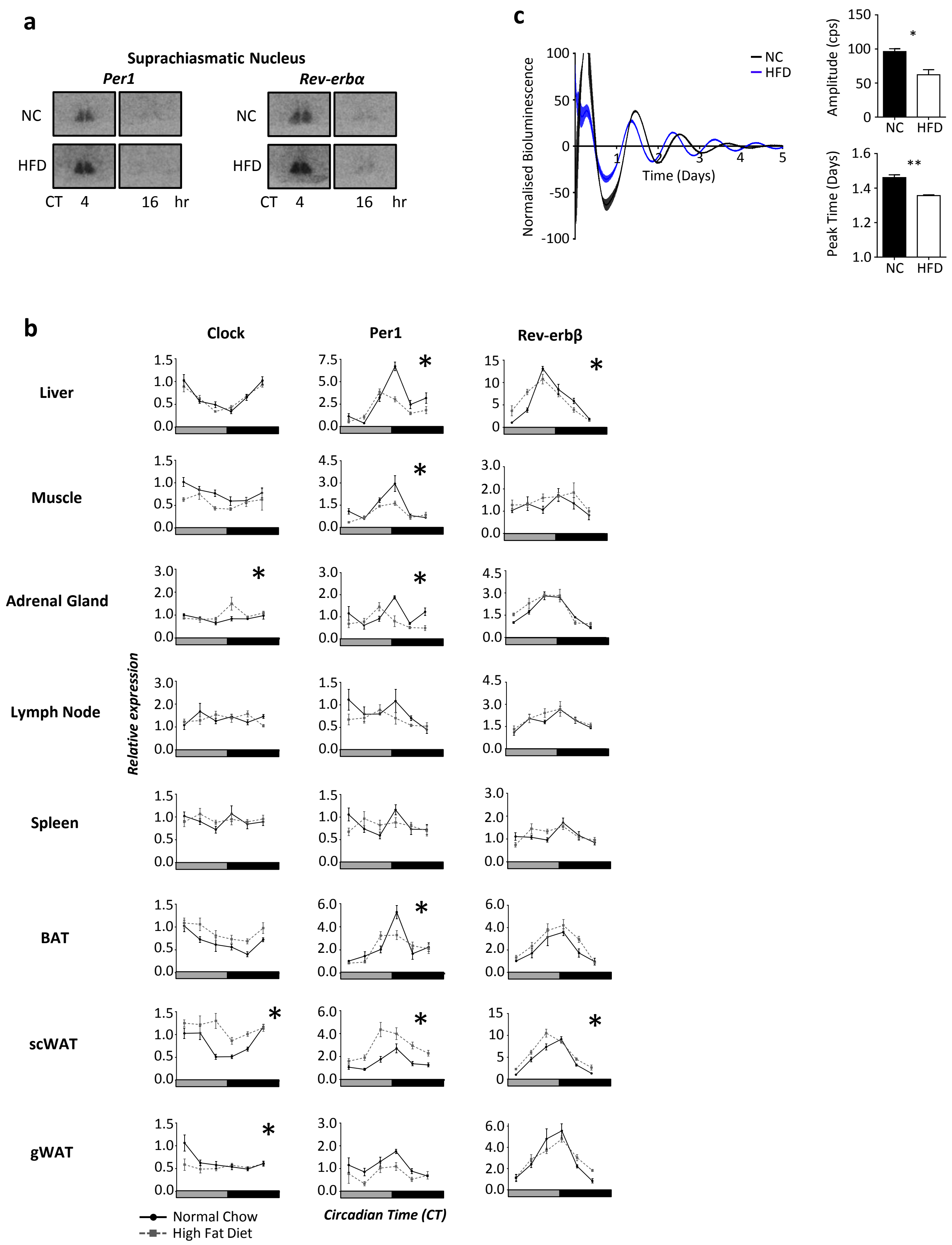

# Supplementary Figure S3

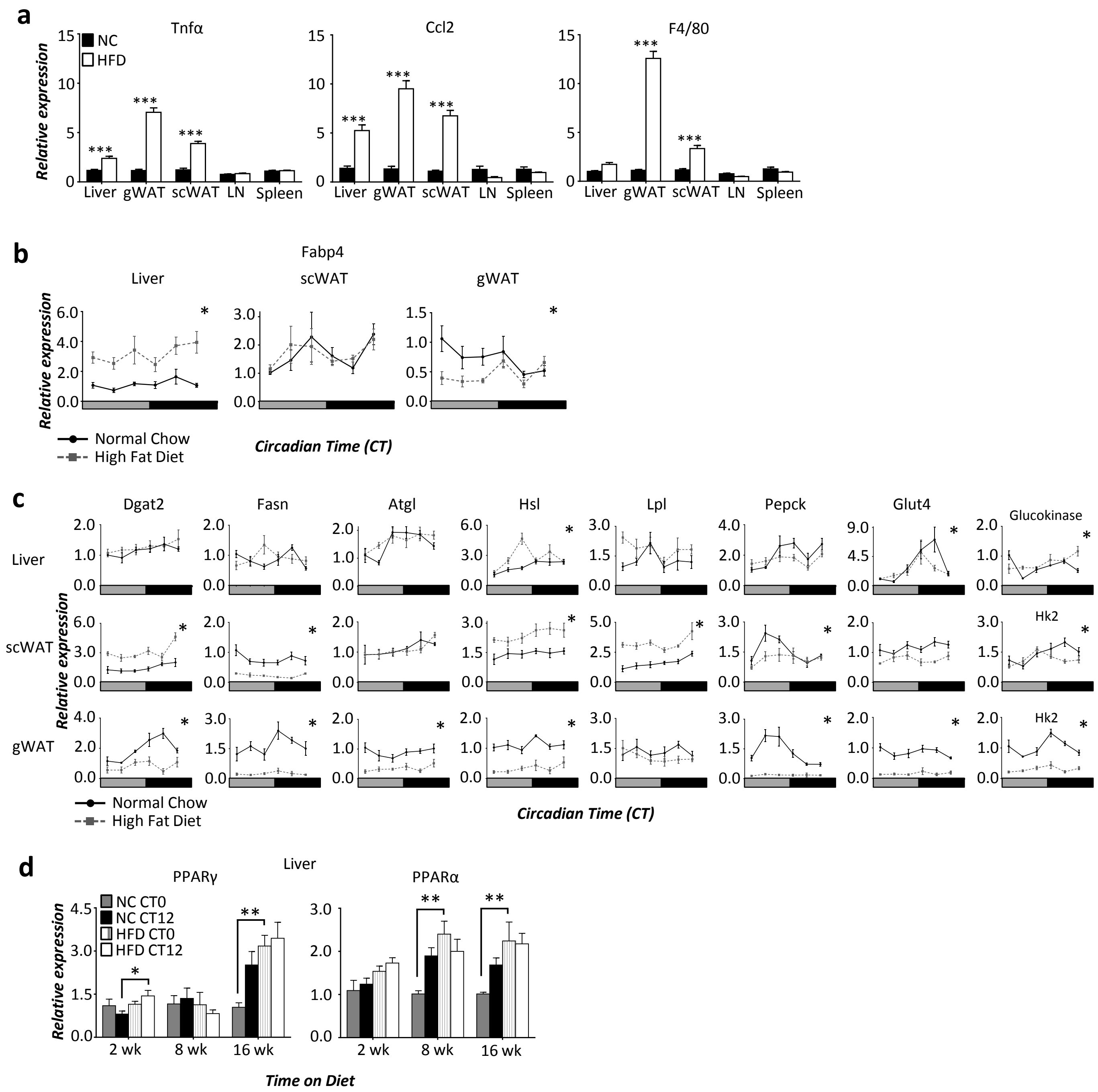

# Supplementary Figure S4

**a**

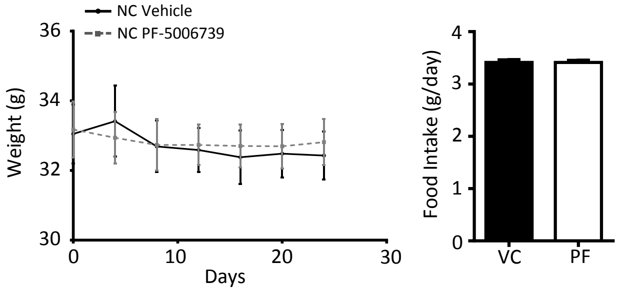


**b**

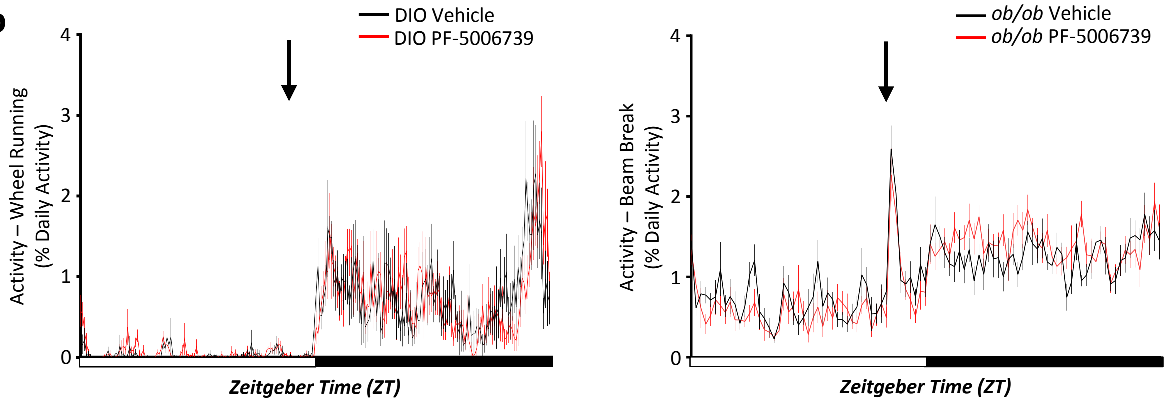


**c**

| DIO mice | Vehicle | PF-5006739 | p value |
|---|---|---|---|
| Fasted Blood Glucose (mmol/L) | 10.4 ± 0.5 | 9.3 ± 0.7 | 0.237 |
| Fasted Insulin (ng/ml) | 6.26 ± 0.89 | 5.04 ± 0.30 | 0.251 |
| HOMA-IR | 72.2 ± 12.1 | 54.0 ± 4.1 | 0.210 |
| Glucose Stimulated Insulin (% Change at 30 min) | 102.4 ± 6.4 | 121.7 ± 12.0 | 0.167 |
| Serum Adiponectin (µg/ml) | 4.26 ± 0.30 | 4.27 ± 0.34 | 0.978 |

| *ob/ob* mice | Vehicle | PF-5006739 | p value |
|---|---|---|---|
| Fasted Blood Glucose (mmol/L) | 16.0 ± 2.5 | 9.7 ± 1.9 | 0.064 |
| Fasted Insulin (ng/ml) | 6.23 ± 0.51 | 9.04 ± 1.28 | 0.064 |
| HOMA-IR | 112.4 ± 22.5 | 91.6 ± 13.9 | 0.445 |
| Glucose Stimulated Insulin (% Change at 30 min) | 77.5 ± 7.2 | 95.1 ± 12.1 | 0.235 |
| Serum Adiponectin (µg/ml) | 2.92 ± 0.24 | 2.74 ± 0.16 | 0.541 |

**d**

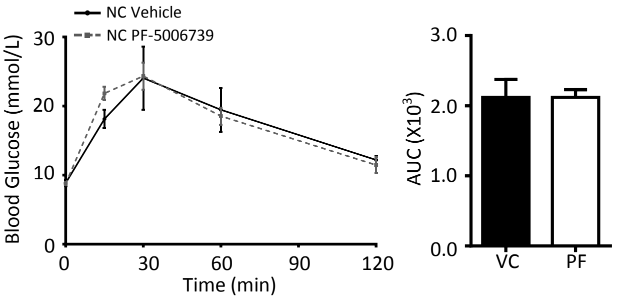

# Supplementary Table S1

## Inhibition and pharmacokinetic parameters of PF-5006739

*Enzyme $IC_{50}$ and whole cell $EC_{50}$ values (values with 95% confidence intervals, CIs)*

| Enzyme | | | Whole Cell | | |
|---|---|---|---|---|---|
| $IC_{50}$ (nM) | | Ratio CK1δ/CK1ε | $EC_{50}$ (nM) | | Ratio CK1δ/CK1ε |
| CK1δ (95% CI) | CK1ε (95% CI) | | CK1δ (95% CI) | CK1ε (95% CI) | |
| 3.9 (2.8-5.3) | 17 (14-20) | 4.4 | 15 (13-19) | 83 (77-99) | 5.5 |

*Pharmacokinetic parameters after sc administration to mice (FVB)*

| ***PF-5006739*** | ***Plasma*** | ***Brain*** | ***Ratio*** | ***Plasma**** | ***Brain**** |
|---|---|---|---|---|---|
| Dose (mg/kg) | 17 | 17 | | 10 | 10 |
| Total $C_{max}$ (ng/mL) | 2650 | 354 | | 1558.8 | 208.1 |
| $AUC_{0\text{-t last}}$ (ng*h/mL) | 3860 (4.0h) | 478 (4h) | | 2269.7 (4h) | 281.0 (4h) |
| $AUC_{0\text{-}\infty}$ (ng*h/mL) | 3990 | 494 | | 2346.1 | 290.5 |
| $T_{max}$ (h) | 0.5 | 0.5 | | 0.5 | 0.5 |
| Protein Binding ($f_u$) | 0.25 | 0.18 | | 0.25 | 0.18 |
| Free $C_{max}$ (nM) | 662.5 | 63.7 | | 389.5 | 37.4 |
| Free $AUC_{0\text{-}\infty}$ (ng*h/mL) | 997.5 | 88.9 | | 586.5 | 52.3 |
| Free $AUC_{0\text{-}\infty}$ $C_b/C_p$ | | | 0.12 | | |
| Free $AUC_{0\text{-}\infty}$ $C_{b,u}/C_{p,u}$ | | | 0.09 | | |
| Free $C_{max}$ $C_{b,u}/C_{p,u}$ | | | 0.10 | | |

* Scaled to 10mg assuming linear PK. (0.588)

*Observed free $C_{max}$ concentration relative to whole cell CK1δ and CK1ε $EC_{50}$*

| | Free Plasma (10mg/kg, SC) | Free Brain (10mg/kg, SC) | Free Plasma (17mg/kg, SC) | Free Brain (17mg/kg, SC) |
|---|---|---|---|---|
| CK1δ | 26.0 | 2.45 | 44.2 | 4.2 |
| CK1ε | 4.7 | 0.45 | 8.0 | 0.77 |

# Supplementary Table S2

## Mouse qPCR primers.

| Gene | Direction | Primer Sequence (5' to 3') |
|---|---|---|
| *18S* | Forward | TCCGACCATAAACGATGCCGACT |
| | Reverse | TCCTGGTGGTGCCCTTCCGTCAAT |
| *Adipoq* | Forward | GCTCCTGCTTTGGTCCCTCCAC |
| | Reverse | GCCCTTCAGCTCCTGTCATTCC |
| *Atgl* | Forward | TGTGGCCTCATTCCTCCTAC |
| | Reverse | GTAACTGGGTAGGCTGCCAT |
| *Bmal1* | Forward | CCAAGAAAGTATGGACACAGACAAA |
| | Reverse | GCATTCTTGATCCTTCCTTGGT |
| *Ccl2* | Forward | GGCTCAGCCAGATGCAGTTAA |
| | Reverse | CCTACTCATTGGGATCATCTTGCT |
| *Cry1* | Forward | TCGCCGGCTCTTCCAA |
| | Reverse | TCAAGACACTGAAGCAAAAATCG |
| *Dbp* | Forward | CCGTGGAGGTGCTAATGACCT |
| | Reverse | CCTCTGAGAAGCGGGCC |
| *Dgat2* | Forward | AGTGGCAATGCTATCATCATCGT |
| | Reverse | TCTTCTGGACCCATCGGCCCCAGGA |
| *Fabp4* | Forward | GAAAACGAGATGGTGACAAGC |
| | Reverse | TTGTGGAAGTCACGCCTTT |
| *Fasn* | Forward | CCCAGAGGCTTGTGCTGACT |
| | Reverse | CGAATGTGCTTGGCTTGGT |
| *F4/80* | Forward | AAGACTTGATACTCCAAAGTGAGC |
| | Reverse | GAAGGAAGCATAACCAAGATCCC |
| *Gk* | Forward | CCCTGAGTGGCTTACAGTTC |
| | Reverse | ACGGATGTGAGTGTTGAAGC |
| *Glut4* | Forward | GTCCTCCTGCTTGGCTTCTT |
| | Reverse | AGCTGAGATCTGGTCAAACG |
| *Hsl* | Forward | GCTGGGCTGTCAAGCACTGT |
| | Reverse | GTAACTGGGTAGGCTGCCAT |
| *Hk2* | Forward | GAAGATGATCAGCGGGATGT |
| | Reverse | TCTGGATTCCGTCCTTATCG |
| *Lpl* | Forward | AGGGCTCTGCCTGAGTTGTA |
| | Reverse | CCATCCTCAGTCCCAGAAAA |
| *Pepck* | Forward | GGCCACAGCTGCTGCAG |
| | Reverse | GGTCGCATGGCAAAGGG |
| *Per2* | Forward | GCCTTCAGACTCATGATGACAGA |
| | Reverse | TTTGTGTGCCTCAGCTTTGG |
| *PPARα* | Forward | GAGGGTTGAGCTCAGTCAGG |
| | Reverse | GGTCACCTACGAGTGGCATT |
| *PPARγ* | Forward | AGGCCGAGAAGGAGAAGCTGTTG |
| | Reverse | TGGCCACCTCTTTGCTCTGCTC |
| *Rev-erbα* | Forward | GTCTCTCCGTTGGCATGTCT |
| | Reverse | CCAAGTTCATGGCGCTCT |
| *Rev-erbβ* | Forward | TCATGAGGATGAACAGGAACC |
| | Reverse | GAATTCGGCCAAATCGAAC |
| *TNFα* | Forward | TCTCTTCAAGGGACAAGGCTG |
| | Reverse | ATAGCAAATCGGCTGACGGT |

## Primers used for generation of *in situ* probes.

| Gene | Direction | Primer Sequence (5' to 3') |
|---|---|---|
| *Bmal1* | Forward | GTAGATCAGAGGGCGACAGC |
| | Reverse | GGGAGGCGTACTTGTGATGT |
| *Rev-erbα* | Forward | AGGGCACAAGCAACATTACC |
| | Reverse | CTGAGAGAAGCCCACCAAAG |

# Supplementary Table S3

## Acrophase analysis by linear harmonic regression.

| Acrophase (CT) | Liver | | | Adrenal Gland | | | Muscle | | | BAT | | |
|---|---|---|---|---|---|---|---|---|---|---|---|---|
| | **NC** | **HFD** | **Advance** | **NC** | **HFD** | **Advance** | **NC** | **HFD** | **Advance** | **NC** | **HFD** | **Advance** |
| **Bmal1** | 22.0 | 20.7 | 1.3h | 21.6 | 18.8 | 2.8h | 0.3 | 23.6 | 0.7h | 23.6 | 23.2 | 0.4h |
| **Clock** | 22.0 | 21.5 | 0.5h | 21.5 | --- | --- | 1.3 | --- | --- | 1.0 | 1.1 | -0.1h |
| **Cry1** | 19.8 | 18.7 | 1.1h | 18.0 | 14.8 | 3.2h | 23.5 | 21.8 | 1.7h | 20.9 | 19.2 | 1.7h |
| **Dbp** | 9.8 | 7.9 | 1.9h | 8.7 | 5.9 | 2.8h | 10.6 | 9.9 | 0.7h | 10.7 | 11.2 | -0.5h |
| **Per1** | 13.0 | 10.6 | 2.4h | --- | 7.6 | --- | 10.8 | 10.8 | 0.0h | 12.3 | 12.3 | 0.0h |
| **Per2** | 14.7 | 12.1 | 2.6h | 13.8 | 12.1 | 1.7h | 12.3 | 12.0 | 0.3h | 12.8 | 13.4 | -0.6h |
| **Rev-erbα** | 7.3 | 4.4 | 2.9h | 7.3 | 5.2 | 2.1h | 5.6 | 4.7 | 0.9h | 7.9 | 7.5 | 0.4h |
| **Rev-erbβ** | 10.0 | 7.9 | 2.1h | 9.3 | 8.0 | 1.3h | 11.1 | 11.8 | -0.7h | 10.2 | 10.5 | -0.3h |
| **Mean Advance** | | | **1.9h** | | | **2.3h** | | | **0.5h** | | | **0.1h** |

--- Indicates no significant rhythm detected by CircWave
